# Investigating the mechanism of Echovirus 30 cell invasion

**DOI:** 10.3389/fmicb.2023.1174410

**Published:** 2023-07-06

**Authors:** Yucai Liang, Junbing Chen, Congcong Wang, Bowen Yu, Yong Zhang, Zhijun Liu

**Affiliations:** ^1^Department of Microbiology, Weifang Medical University, Weifang, China; ^2^Key Laboratory of Carcinogenesis and Translational Research (Ministry of Education), Gastrointestinal Cancer Center, Peking University Cancer Hospital and Institute, Beijing, China; ^3^Department of Immunology, Weifang Medical University, Weifang, China; ^4^National Institute for Viral Disease Control and Prevention, Chinese Center for Disease Control and Prevention, Beijing, China

**Keywords:** E30, invasive mechanisms, enterovirus, aseptic meningitis, central nervous system

## Abstract

Viruses invade susceptible cells through a complex mechanism before injecting their genetic material into them. This causes direct damage to the host cell, as well as resulting in disease in the corresponding system. Echovirus type 30 (E30) is a member of the Enterovirus B group and has recently been reported to cause central nervous system (CNS) disorders, leading to viral encephalitis and viral meningitis in children. In this review, we aim to help in improving the understanding of the mechanisms of CNS diseases caused by E30 for the subsequent development of relevant drugs and vaccines.

## 1. Introduction

Enteroviruses (EVs) are members of both the small RNA virus family and the enterovirus family; they primarily consist of four groups of EVs (EV-A, -B, -C, and -D) and three groups of Rhinoviruses (RV-A, -B, and -C) (Caroline et al., 2013). Viral protein 1 (VP1) sequences correlate strongly with serotype and can therefore be used to classify EVs and discover novel genotypes and EVs strains (Oberste et al., 1999). Newly isolated EVs have been given the serial number-based names EV-D68, EV-B69, EV-D70, and EV-A71. Although most EVs infections are asymptomatic, there are more than 20 clinically recognized disorders associated with many EVs types, with millions of people being infected annually (Caroline et al., 2013; Hoang et al., 2019). Clinically, EVs infections can cause a variety of symptoms, such as hand-foot-and-mouth disease (Zhang et al., 2010; Huang et al., 2018), acute hemorrhagic conjunctivitis (Leveque et al., 2010), poliomyelitis (World Health Organization Country Office Tajikistan et al., 2010; Hall et al., 2019), encephalitis (Worner et al., 2021), and aseptic meningitis (AM), while cardiac illness may ultimately result in death (Caroline et al., 2013; Zell et al., 2017; Del Cuerpo et al., 2022). Over 90% of cases of viral meningitis cases are caused by EVs, while CNS involvement is frequently associated with systemic infections, liver cell death, and myocarditis (Caroline et al., 2013). Interstitial laxity and edema with a small amount of erythrocyte infiltration in the brain parenchyma have been found to be associated with neurological symptoms in an *in vitro* mouse E30 infection model (Li et al., 2022). However, studies on pediatric patients have revealed that children with AM following an E30 infection frequently experience transient regional blood flow decreases, which may be the result of cerebrovascular inflammation (Nishikawa et al., 2000). Infected patients may also experience severe migraines, high fevers, photophobia, and other clinical manifestations that cause them great suffering. Although the CNS is protected by a complex barrier, viral pathogens can establish infections in cerebrovascular endothelial cells, causing severe damage to the barrier, reaching adjacent glial cells and neurons, causing systemic complications, and, in the most severe cases, causing death (Wright et al., 2019; Del Cuerpo et al., 2022). To date, E30 has become a global epidemic, therefore inflicting a major burden on families around the world (Brunel et al., 2008; Baek et al., 2011; Kim et al., 2012; Takamatsu et al., 2013; Ramalho et al., 2019; Benschop et al., 2021; Del Cuerpo et al., 2022). E30 is the most frequently isolated EVs strains in EV-B and is a prevalent cause of the current outbreak of viral meningitis, of which children and immunocompromised individuals are the clinical categories with the highest occurrence rates. During the first few weeks of life, neonates and infants have the highest risk of developing severe EVs-related illnesses, with vertical transmission possibly occurring before or during birth owing to maternal infection (Haston and Dixon, 2015; Chuang and Huang, 2019). Furthermore, infection with intrauterine EVs (early and late pregnancy) has also been linked to fetal death (Tassin et al., 2014). Although E30 has caused multiple outbreaks around the world, little is known about its reproduction cycle (Vandesande et al., 2020). Considering that viral invasion is a crucial step in infection, it is essential to examine this process. We applied cryo-electron microscopy (EM) to visualize the structure of E30, identified its related receptors, and then investigated the molecular mechanisms of receptor recognition (Wang et al., 2020). This provides a better understanding of how E30 spreads and therefore provides a basis for the creation of antiviral medications and vaccines.

## 2. Biological characteristics of E30

E30 is one of the important pathogenic agents of AM, a clinically common bacterial culture-negative meningitis of the cerebrospinal fluid (CSF) that results in headaches, nausea, and vomiting (de la Motte et al., 2018). In addition, the high rate of E30 separation in patients with acute resting palsy (AFP) and hand-foot-oral disease presents a novel problem, while the evolution of E30 has exhibited links with the global growth in the incidence of type 1 diabetes (Paananen et al., 2007; Tao et al., 2014; Ivanova et al., 2019; Bubba et al., 2020). Considering this, an extensive investigation into the fundamental mechanics of E30 is necessary. Symptoms of AFP in immune-suppressed transplant recipients and acute myalgia and rhabdomyolysis outbreaks in Brazil have revealed serious non-neuropathic findings and also increased the diversity of clinical symptoms induced by E30, which should be considered in future research (Sousa et al., 2019; Mauri et al., 2020).

**Figure 1:**
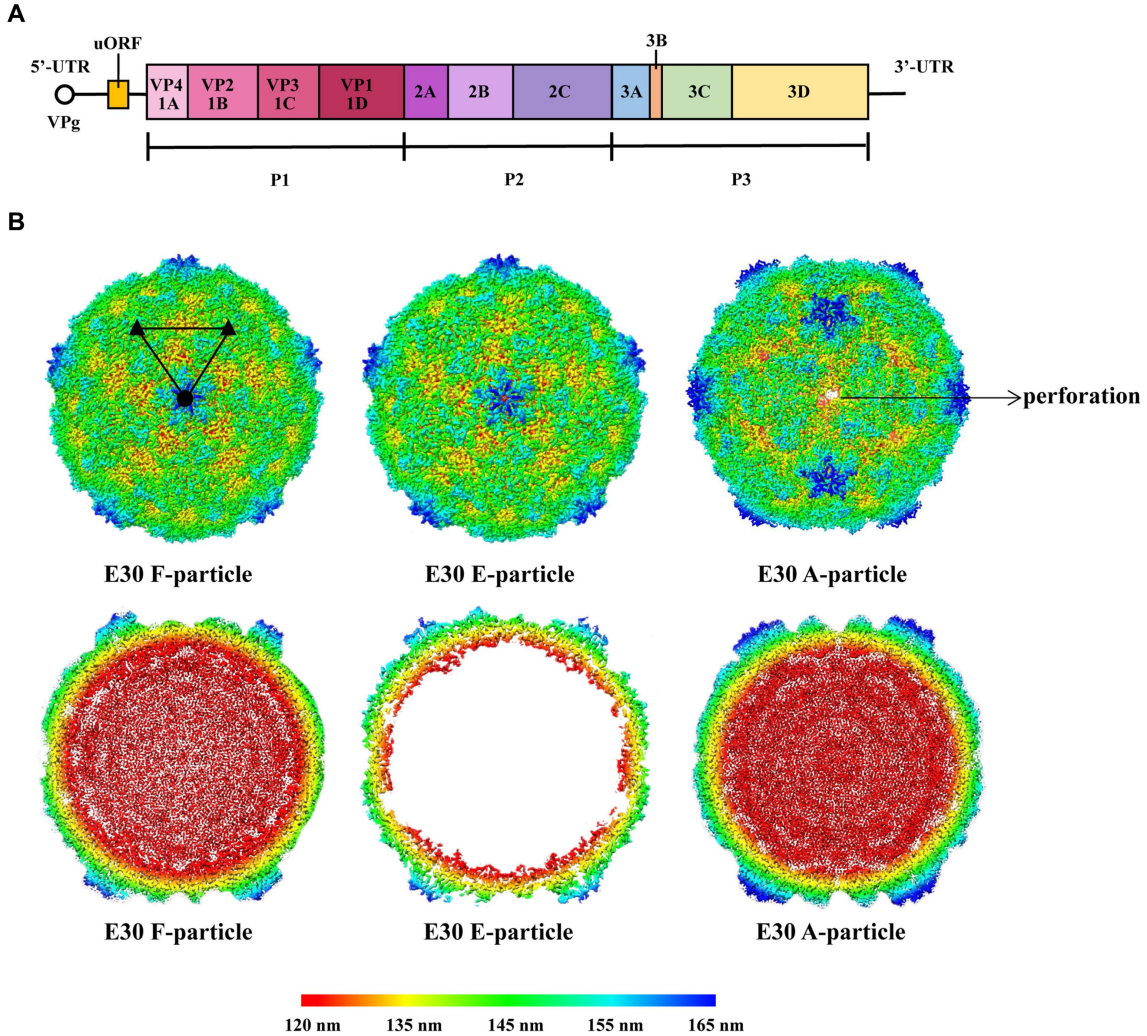
**(A)** Schematic representation of the E30 gene structure. The uORF is on the left. The 5’ end of the E30 genome is covalently linked to a viral protein genome-linked (VPg). **(B)** The first line indicates the surface of E30 F-, E-, and A-particles along the icosahedral twofold axes. The A-particle forms perforations at the icosahedral two-fold axes. The surfaces are rainbow-colored by radius from red through indigo, green, and yellow to blue, which correspond to 120, 135, 145, 155, and 165 nm. The second line represents thin slices of the central sections of each particle; they use the same color scheme.

**Figure 2:**
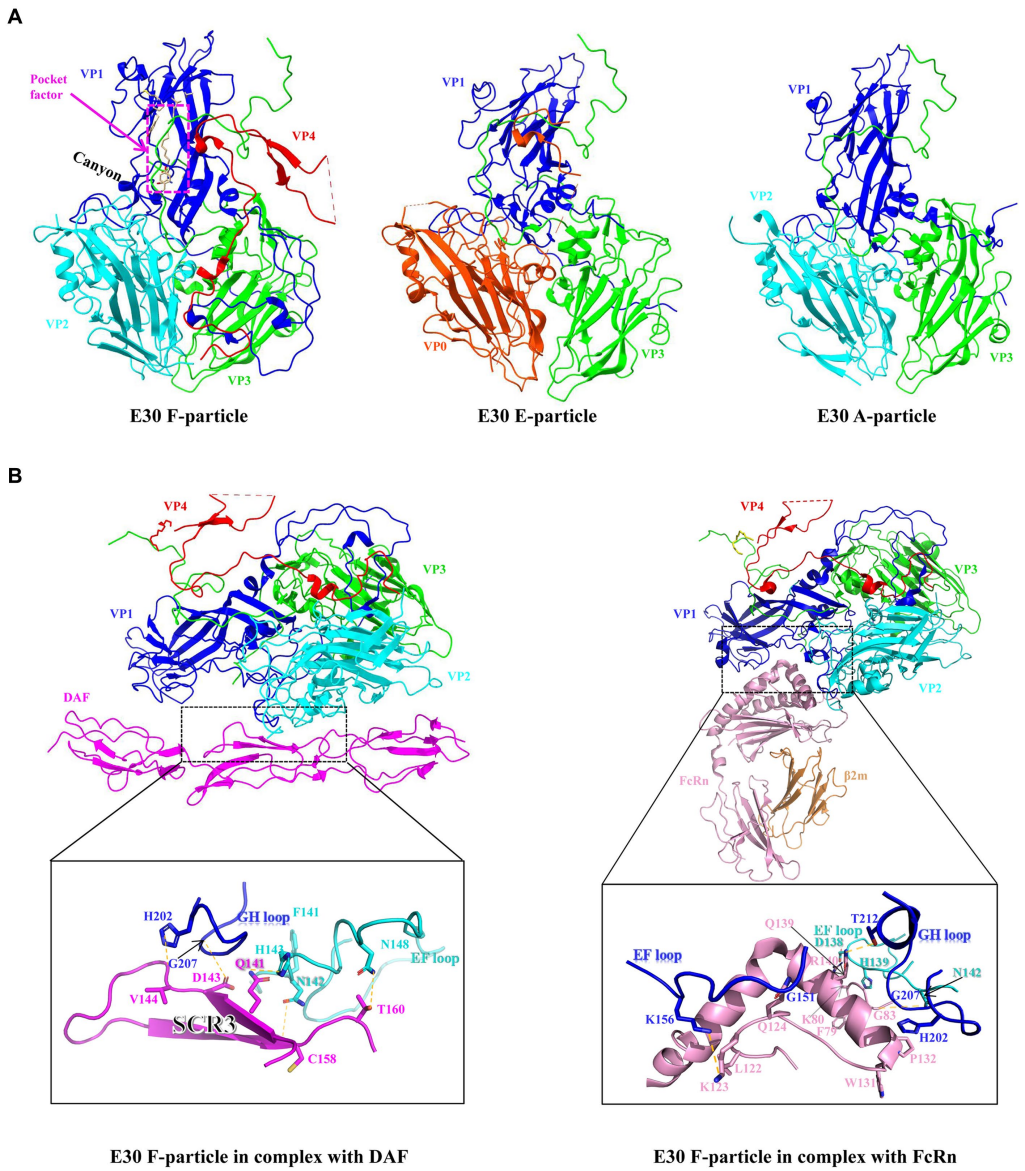
**(A)** Surface of the protein structures of E30 F-, E-, and A-particles (VP0, orange red; VP1, blue; VP2, indigo; VP3, green; VP4, red), The F-particle has a hydrophobic pocket containing a pocket factor in VP1, labeled with magenta. And the canyon region is in VP1. **(B)** Residues at the main E30-DAF (left) and E30-FcRn (right) interfaces. The side or main chains involved in mutual interactions are shown as sticks. The hydrogen bonds are shown as yellow dashed lines.

As a member of the EV-B family, E30 is similar to the majority of EVs in that the virus particle is icosahedral and generally 24–30 nm in size. The genome consists of a 7.5-kb single-strand positive-chain RNA that encodes P1 structural proteins (VP1, VP2, VP3, and VP4), in addition to P2 (2A, 2B, and 2C), and P3 (3A, 3B, 3C, and 3D) (Caroline et al., 2013; Figure 1A). VP1-3 is found on the surface of the capsid, while VP4 is present within it. Beneath VP1 is a hydrophobic pocket, which is believed to affect the stability of the virus (Marjomaki et al., 2015; Figure 2A, left). In addition, at the 5′- terminal and 3′-terminal ends of the RNA molecule is a non-coding region that contains RNA-acting elements essential for RNA replication and translation. The 5′-untranslated region (5’-UTR) contains several domains, including some that are essential for RNA replication, including OriL and translational regions such as internal ribosomal entry sites (IRESs) (Ogram and Flanegan, 2011; Lin et al., 2021). Many EVs have been shown to have an upstream open reading frame (uORF), and we discovered that E30 has this structure as well by protein sequence comparison (Figure 1A). The uORF is hypothesized to aid virus development in gut epithelial cells and is essential for viral replication (Guo et al., 2019; Lulla et al., 2019). Furthermore, the Poly A tail is situated after the 3′-UTR (OriR). Under cryo-EM, E30 exhibits three stages, namely natural empty particles (without RNA, termed “E-particles”), uncoated intermediates (termed “A-particles”), and mature virions (termed “F-particles”), with different lifetimes (Figure 2A). The sedimentation coefficient of F particles in E30 (~150 S) is substantially lower than that of mature virus particles in most EVs, despite the possibility that most EVs have comparable structural characteristics. This suggests that F particles may be combined with a low ratio of A particles (Wang et al., 2020). VP1–3 is on the surface of the capsid, while VP4 is present under it. Under VP1 is a hydrophobic pocket, which is believed to affect the stability of the virus. The capsid proteins (VP1, VP2, and VP3) have conventional β-stranded reverse-parallel barrel structures, within which the N terminus is contained, while the C terminus is on the surface. F-particles have a hydrophobic pocket in VP1 that contains pocket factors, whereas E and A do not. The high immunogenicity of granules E and F, alongside a substantial expansion of granule A, represents the structural movement of the particles, and perforations can occur on the bi-folded axis of the icosahedral body (Figure 1B, upper right). Both E and F are generally “closed” and have highly organized and identical surface features, suggesting that both could be exploited for potential vaccine development. Notably, when comparing the structure of E30 to that of other members of the EVs, the VP1 BC loop appeared highly differential, which indicated that it could be an important structural signature for different EVs serotypes. In addition, the VP1 GH and VP2 EF loops are both relatively well conserved, indicating that they can be rationally targeted to obtain broad neutralizing antibodies against most EV-B (Wang et al., 2020). The gene structure, virus protein structure, particle structure, and the complex structure with receptor antibody of E30 are shown in Figures 1, 2.

## 3. The invasion process of E30

This is a comprehensive review of the mechanism by which E30 invades cells: it involves the whole viral replication cycle. The viral replication cycle consists of adsorption, penetration, uncoating, biosynthesis, and assembly, including the fundamental steps of maturation and release. The length of the replication cycle varies by virus type, with the first three steps being most closely associated with the invasion process. Asymptomatic carriers of E30, one of the most frequently detected EV-B serotypes, primarily transmit the virus via the fecal route (Brunel et al., 2008), or via respiratory or pharyngeal mucosal infection (Wolthers et al., 2008; Harvala and Simmonds, 2009). Although most enteric viruses are delivered via the fecal channel, they rarely induce gastrointestinal symptoms, since their primary target organs are the neural, muscular, or other systems. This has been well reported for E30, which exhibits tropisms for both brain and muscle tissues (Li et al., 2022). Although a recent study demonstrated that enteric viruses like Norovirus can effectively and continuously infect and replicate in the salivary glands, no study has demonstrated that E30 can cause nervous system diseases via the salivary glands; therefore, additional testing may be necessary to confirm this pathway (Ghosh et al., 2022). To date, the majority of studies have demonstrated that the BBB is the primary entry point for E30 into cerebral tissue. In addition, E30 may infiltrate the brain via lymphatic tissue, causing severe diseases of the CNS (Wiatr et al., 2020). During meningitis caused by E30 infection, viruses may interact with two major CNS barriers: the blood–brain barrier (BBB) and the blood cerebrospinal fluid barrier (BCSFB), while E30 infection has been shown to increase the migration of naïve T cells, including CD3^+^, CD4^+^, and CD8^+^, in the BCSFB (Dahm et al., 2018; Wiatr et al., 2019). When the susceptible population is infected with E30, the virus invades target cells through a complex array of mechanisms, and since its associated receptors are expressed in many cell types, E30 can infect numerous cell types throughout the body, e.g., in fecal transmission, possibly targeting human umbilical vein primary endothelial cells or gastrointestinal epithelial cells (Ylipaasto et al., 2010; Morosky et al., 2019).

Echovirus exhibits cell type selectivity and preferentially infects intestinal cells to breach the intestinal barrier in primary human fetal enteroid cells (Drummond et al., 2017); *in vitro* studies on human rhabdomyosarcoma cells (RD cells) (Tiwari and Dhole, 2017) and human neuroblastoma cells (SK-N-SH cells) (Lee et al., 2012) have previously demonstrated that the activation of TrioGEFD2 results in the production of intracellular nitric oxide, which is then amplified by E30 infection, resulting in the death of neuronal cells. In addition, enteroviruses generally replicate in lymphoid tissue associated with the gastrointestinal system. To infiltrate tissues at other secondary replication sites, viruses must survive in the circulatory system and then discover paths through endothelial cells to transcend the physical barrier between blood and tissue. E30 may be able to accomplish this step by infecting vascular endothelial cells (Ylipaasto et al., 2010).

### 3.1. Adsorption

The first step of E30 invasion is adsorption, which mostly consists of the virus attaching itself to the surface of cells that can be infected. One prominent feature of Picornaviridae is the diversity of the receptors they use, since various receptors are known and have been shown to facilitate the invasion of EVs, including both the adsorption receptors and the uncoating receptors (Harvala and Simmonds, 2009). E30 invasion mainly depends on specific adsorption, i.e., the binding of virus attachment protein (VAP), a viral surface adsorbent protein, to specific receptors on the host cell surface. Even though the majority of CV-B members in EV-B use the coxsackievirus-adenovirus receptor (CAR) to connect to and enter host cells, studies on the early invasion mechanisms of E30 have shown that it preferentially clings to DAF (decay-accelerating factor, accelerated decay factor, CD55) (Vandesande et al., 2020). For EV-B, DAF, a glycosyl-phosphatidyl ionositol (GPI)-anchored membrane glycoprotein, is considered to be an important adsorption receptor for the initial invasion of E30 into cells.

**Figure 3:**
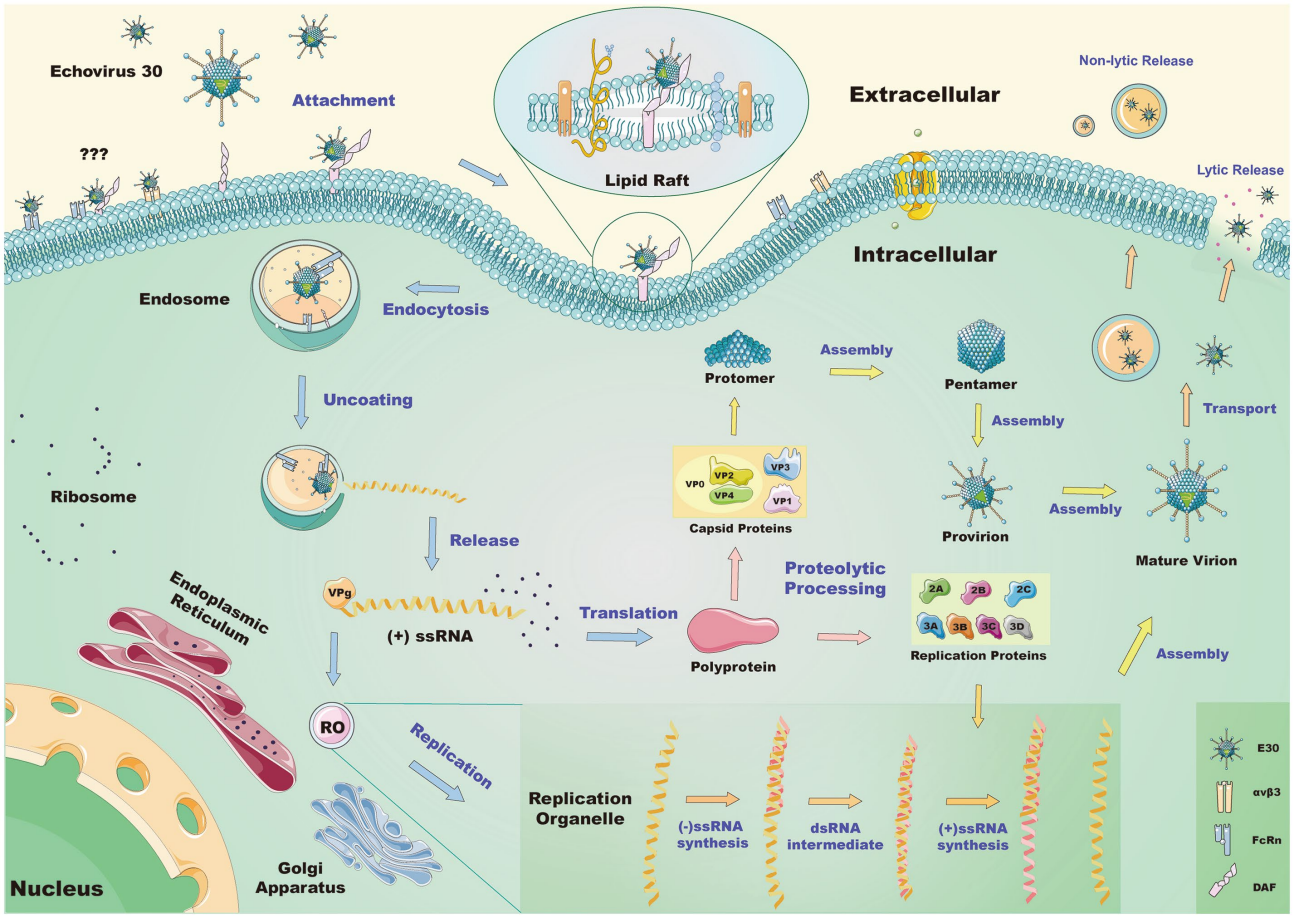
Schematic of the mechanism by which E30 invades the RD cell. Lipid raft proteins mediate endocytosis when E30 interacts with DAF on the surface of the cell. The queried pathway suggests it is unclear whether αvβ3 or FcRn alone or in association with DAF mediates the same process. After entering the cells, E30 decapitates in the endosome via FcRn, causing conformational changes in the virus and the release of genetic material. This genetic material is then replicated by the replication organelle (RO), where the translation polyprotein further hydrolyzes to a replication protein and capsid protein. Of these, the former may participate in the replication process, and the latter further processes and assembles the genetic material into new virions, which are released from the cells in a lytic or non-lytic pathway.

DAF is located on the cell surface of endometrial epithelial and mesenchymal cells, while its expression is regulated by estrogen and progesterone (Wang et al., 2018). Previous cases have confirmed that intrauterine E11 infection can lead to congenital hypogonadism, but there are few instances of maternal EVs infection and its connection with fetal pathogenesis during pregnancy, however, it is uncertain if E30 can cause intrauterine infection or fetal infection of endometrial stromal cells via DAF (Tassin et al., 2014). The region of the enterovirus particle that binds to DAF is vividly referred to as a “canyon”. Numerous virus receptors are believed to bind to the “canyon”, a depression located near the fivefold axes on the surface of the capsid of EVs. E30 contains the canyon-like depression; there are five distinct ~15 Å deep depressions. A ridge between each depression is formed by the C-termini of VP1 and VP3 (Figure 2A, left). The short common repetitive sequence SCR3 in DAF interacts with the VP1 GH and VP2 EF loops in E30 via water-affinity interactions, according to structural studies (Wang et al., 2020; Figure 2B, left). Despite structural biology studies that have demonstrated that E30 specifically adsorbs DAF well, some evidence indicates that small interfering RNA (siRNA) knockdown has no effect on E30 infection, implying that, despite the fact that DAF promotes E30 binding to target cells, it may not be necessary (Vandesande et al., 2020). In addition to DAF, E30 may have another adsorption receptor, αvβ3 in the integrin family (Ylipaasto et al., 2010). However, it is unclear whether this receptor has the same effect on RD cells (marked by the upper-left question mark in Figure 3). In this study, the properties of these two receptors were examined (Table 1), and neither were shown to have altered the conformation of viral particles. In addition, two additional cytokines, beta2 microglobulin (β2-MG) and the GPI-anchored protein CD59, have been identified as co-factors of echovirus-invasive cells, possibly related to the FcRn receptors described below, which appear to function prior to the formation of A granules following DAF attachment (Goodfellow et al., 2000; Chevaliez et al., 2008). It has also been shown that heparin acetyl sulfate acts as an adsorptive receptor for E5, E6, and Coxsackieviruses (CVs) (Goodfellow et al., 2001; Israelsson et al., 2010). However, it is unknown whether it has the same effect on the E30 invasion of host cells.

**Table 1:** Biological characteristics of E30 adsorption receptors.

Item	DAF	αʋβ3
Related mechanism	Regulates complement activation by binding to and accelerating the decay of convertases, then protects self-cells from complement attack (He et al., 2002; Riabi et al., 2014); DAF binding is facilitated by 138D, 234Q, or 114 K (Goodfellow et al., 2005; Riabi et al., 2014); Echovirus serotypes 6, 7, 11, and 30 mediate virus adsorption by binding to distinct regions of DAF (He et al., 2002; Sobo et al., 2011; Zhao et al., 2019; Wang et al., 2020)	An important integrin for E30 and E32, although it does not contain the RGD (arginine-glycine-aspartic acid) sequence (LaFlamme and Auer, 1996; Yang et al., 2019)
Downstream pathways	In CVBs, Abl and Fyn signals are immediately activated by DAF crosslinking (Coyne and Bergelson, 2006); EV7 entrance is dependent on dynamin and clathrin (Kim and Bergelson, 2012); and E30 invades with the assistance of the lipid raft protein pathway (Vandesande et al., 2020)	Dependent on lipid rafts (Ylipaasto et al., 2010); extracellular signals are transmitted to cells via αʋβ3, possibly causing Ca^2+^, Pyk2, and PI-3 kinase levels to change (LaFlamme and Auer, 1996; Yang et al., 2019)
Structural composition	Four short consensus repetitions make up the DAF functional region (SCR1 to 4) (Williams et al., 2003)	Both sub-submissions have a larger hydrophobic, film domain, and a smaller membrane interior domain composition, joined by non-covalent bonds to produce heterogeneous dicent molecules. The crystal structure of the exterior domain is curved and bent-knee-like (Xiong et al., 2001)

### 3.2. Penetration

When E30 binds to target cells via DAF, its nucleocapsid enters the cytoplasm through the cell membrane. Initial attachment of EVs to the cell surface is followed by endocytosis, whereby the release of genetic material from the deciduous capsid typically occurs. Endocytosis is used here to represent all mechanisms through which cells import fluids, solutes, ligands, plasma membrane components, and granules through membrane invagination and vesicle rupture. In addition to the processes involving attachment to the cell surface, receptor recruitment, and activation of the signaling pathway, there are also the processes of endocytic vesicle formation, the delivery of viral genetic material to the endocytosis compartment, and the classification and escape to the cytoplasmic medium.

As previously reported, several EVs, including E7, may be endocytosed through the clathrin route (Kim and Bergelson, 2012). *In vitro* studies of E30 have revealed that clathrin and dynamic protein-mediated endocytosis are suppressed without affecting the ability of E30 to alter the barrier function of human immortalized brain choroid plexus papilloma cells (Wiatr et al., 2020); Additional research has indicated that E30 may follow the macropinocytosis pathway as opposed to the clathrin pathway (Vandesande et al., 2020). In addition, it has been previously established that E30 enters cells via caveolar/raft endocytosis, since mycomycin, a cholesterol-chelated and lipid raft-disrupting drug, prevents its infection (Ylipaasto et al., 2010).

Thus, the whole process of endocytosis involves E30 binding to a cell surface-related receptor to form a complex, with this substrate becoming the fossa where the plasma membrane ruptures and viral particle ingestion occurs. However, the integrin family may not be involved in the recycling process. E30 can enter BCSFB cells via a lipid raft, thereby triggering endocytosis and the formation of the fossa on the lipid raft. E11, E25, and others depend on the lipid raft process for entry into the cellular compartment of the green monkey kidney cell line, thus indicating the importance of the lipid raft structure in the invasion mechanisms of E30 (Ylipaasto et al., 2010). *In vitro* studies evaluating the effect of E30 infection on the lipid raft protein composition in BCSFB have revealed variations in protein abundance related to cell adhesion, cytoskeletal remodeling, and internalization, and vesicle sprouting under E-30 infection versus uninfected conditions revealed differential abundancy (Wiatr et al., 2020).

### 3.3. Uncoating

After entering the cytoplasm of the host, E30 further degrades the capsid and releases nucleic acid. Likewise, the analogous receptor and the decidual receptor mediate this process. These findings indicate that FcRn may be a common receptor for the novel EV-B virus in neonates. This could also be applied to the echovirus, CV-A9, and other viruses (Zhao et al., 2019). According to previous studies, the CAR also aids certain enteroviruses when entering cells, and Table 2 compares the biological characteristics of FcRn and CAR. However, evaluating the attachment propensity of E30 to the receptor in radioactivity E30 receptor binding assays revealed that E30 is preferentially attached to DAF rather than CAR (Vandesande et al., 2020), but that DAF expression does not sensitize non-permissive cells to infection, suggesting that FcRn acts as the major surface receptor.

**Table 2:** Comparison of the characteristics of two uncoating receptors.

Item	FcRn	CAR
Synthetic gene	FCGRT, a 14-kb gene found on the long arm of chromosome 19, encodes FcRn (Dalakas and Spaeth, 2021)	CAR is encoded by the CXADR gene, which is located on chromosome 21q21.1 and contains 8 exons spanning ~80 kb of genomic DNA (Excoffon, 2020)
Structural composition	β2-microglobulin is an MHC class I-related protein that is noncovalently coupled with a unique transmembrane H chain (Grevys et al., 2015; Baldwin et al., 2019)	CAR has two Ig-type domains situated at the extracellular surface. The protein consists of an N-terminal half, a single membrane-spanning helix, and a C-terminal cytoplasmic tail (Freimuth et al., 2008)
Binding site	The FcRn-binding region on E30 is composed of residues from the VP1 BC, VP1 EF, VP1 GH, and VP2 EF loops; and residues D86 and E87 in the VP1 BC loop, along with residue D138 from the VP2 EF loop, establish charge interactions with FcRn residues K80, R140, and K150 (Wang et al., 2020; Figure 2B)	The interactions between CAR and the virion primarily involve the VP1 BC, VP1 EF, and VP1 GH loops, as well as the VP2 EF loop; the AA’ sheet of CAR interacts with the EF loop of VP2 (N138, N139, and K166) at the canyon’s southern, while the BC loop of CAR penetrates deep into the canyon (Freimuth et al., 2008; Xu et al., 2021)
Downstream pathways	It may be concerned with the related pathway of type I interferon (IFN) (Sun et al., 2022)	HAdV-C5 FK (FKAd5) interacts with CAR and activates p44/p42 MAPK and JNK, as well as the transcription factor NF-κB in human lung epithelial cells (Tamanini et al., 2006)

FcRn is an MHC-class protein composed of a heavy chain encoded by the FCGRT gene and a light chain 2-MG encoded by the 2 M genes. Its alpha chain consists of three distinct domains (α1, α2, and α3). The α3 and β2m domains are then folded into structures that resemble immunoglobulin (Ig) (Oganesyan et al., 2014). Contrary to the receptor-linked DAF, the FcRn receptor binds to the “canyon region” of the viral particle via its FCGRT subunit (a deep depression around fivefold axes). Under acidic conditions, the binding of FcRn to E30 leads to the efficient release of “pocket factor” and commences the structural change of the viral particle (Zhao et al., 2019). By analyzing the structures of E30 and FcRn, it has been determined that FcRn enters the E30 canyon depression largely via the α2 and α3 helices (Wang et al., 2020). The FcRn binding sites on E30 contain antigen residues from the VP1 BC, VP1 EF, VP1 GH, and VP2 EF loops, which make up the valley’s north and south sides (Wang et al., 2020). FcRn is extensively expressed in the cytoplasm, most likely on the endosomal membrane, and also has an adsorption role on the cell surface. DAF as the first attachment receptor (Zhao et al., 2019), E30 transfer to FcRn on the cell membrane, and FcRn-mediated virus uncoating, may all be involved in the entire process. However, in the study of E6, there may be a process of FcRn-mediated endocytosis on the cell membrane surface, and it is unclear if FcRn has the same impact on E30, or if it instead works in conjunction with DAF to facilitate the entry of E30 into cells (Zhao et al., 2019) (the marker at the upper left question mark in Figure 3). It has been shown that FcRn is highly expressed in the colon, kidney, liver, lung, placenta, hematopoietic cells, vascular endothelium, and blood–brain barrier, while the distribution of its expression corresponds with the histological tropism of E30 (Roopenian and Akilesh, 2007). The virus spreads from the gastrointestinal tract to other organs or systems, such as the CNS, liver, spleen, lungs, bone marrow, and heart, resulting in a variety of disorders (Zhao et al., 2019). As a functioning receptor, FcRn may allow E30 to traverse the blood–brain barrier and the blood-placental barrier (Zhao et al., 2019).

Human FcRn has been demonstrated to be a two-in-one attachment-uncoating receptor of E18, and we hypothesize that this receptor plays a similar role in E30 invasion, because siRNA knockdown of DAF alone does not prevent E30 invasion, but siRNA treatment of FcRn completely abolishes E30 infection, as previously mentioned (Vandesande et al., 2020; Chen et al., 2022). Although FcRn plays an important role in E30 viral decapitation and is a necessary receptor for E30 invasion, studies have demonstrated that human FcRn expression is insufficient to infect echovirus through an *in vivo* pathway of the gastrointestinal tract; Type I IFN is a major driver of echovirus transmission from the gastrointestinal tract to secondary sites, including the liver and pancreas; and Type III IFN inhibits persistent echovirus infection in gastrointestinal epithelial cells (Wells et al., 2021, 2022). Numerous genes associated with the IFN pathway have been found to be substantially up-regulated in infected brain tissue of suckling mice, and these genes play a crucial role in the earliest stages of host cell infection (Sun et al., 2022). To investigate the mechanism of action of IFN in E30 infection, more research is required.

According to one previous study, changes in monocationic and divalent cations promote EVs capsid opening and genome release (Ruokolainen et al., 2019). A combination of low Na^+^, low Ca^2+^, and high K^+^ has been shown to activate the uncoating process in the presence of Mg^2+^ while accelerating the process in the presence of albumin. This could have been because albumin is one of the ligands for FcRn. In addition, the report indicated that the right combination of ions can increase the efficiency of RNA release (Ruokolainen et al., 2019). It remains unclear whether this behavior will be identified at E30 uncoating. Acidic pH values that interact with or expose the receptor cause conformational changes in the virion particles into the active state characteristic of VP4 release from the particles characterized by increased particle size, decreased area of contact between tetrameric aggregates of capsular proteoglycans, and the appearance of the N terminus of the VP1 subunit (Levy et al., 2010; Wang et al., 2012; Ren et al., 2013). Diverse enterovirus granules have twice as many icosahedral symmetry axis apertures (5 × 10 Å) or five times as many openings (up to 8 Å in diameter) as other granules (Wang et al., 2012; Shingler et al., 2013). It has been hypothesized that the primary function of these pores is to release the viral genome (Bostina et al., 2011; Seitsonen et al., 2012; Shingler et al., 2013).

The imaging of E18 virus particles during genome release by electromagnetic freezing revealed that the released genome lacks a portion of the capsid and that the absence of a capsid prototype of the pentamer, called an open particle, forms a hole in the capsid 120 Å in diameter. This is sufficient to allow the release of viral RNA, even if the genome contains a double-stranded RNA fragment (Seitsonen et al., 2012). The detection of open particles in E30 suggested that E30 may be equivalent to the release of the E18 genome, which requires capsid opening and the loss of up to three surrounding capsid proteins (Buchta et al., 2019). The genome is released by an enormous opening in the capsid without the need to unwind its double-stranded RNA components. The approach of viral genomic release could then be applied to the development of antiviral drugs. This step closes the genomic E30 excapsid-releasing region, and the subsequent phase of the invasion process then occurs.

### 3.4. Biosynthesis and assembly

As viral nucleic acids are released into the cytoplasm by uncoating, the genome immediately enters the biosynthesis phase, supplying the host cell with a substantial amount of low-molecular-weight material alongside energy for the synthesis of offspring viral nucleic acids and proteins. We believe that after infection, E30 invades the CNS and replicates in the corresponding cells, further destroying the barrier and neuronal cells, resulting in severe injury to the CNS. After E30 infection, the differentially expressed genes have been shown to be rich in pathways for the negative regulation of neurogenesis, nervous system processes, and neural crest cell migration. In addition, GABRD, GABRB3, GABRE, and GABRQ, which are associated with neuropathy and play an essential role in the growth and migration of neuronal cells, are substantially down-regulated following infection (Sun et al., 2022). There are also data indicating that, after infection, the neuronal differentiation and the related genes of glial cells are down-regulated, suggesting that E30 infection may affect abnormal cellular mitochondrial metabolism to promote the associated apoptosis, which plays a crucial role in the development of the CNS (Sun et al., 2022). In experiments involving both barriers, it has already been demonstrated that infection with E30 can damage junctions, thereby destroying the BBB and the BCSFB (Volle et al., 2015; Dahm et al., 2018). All positive-strand RNA viruses must replicate in a separate membrane replication organelle (RO) (Nchoutmboube et al., 2015). According to previous research, EVs use the endoplasmic reticulum (ER) and Golgi membranes to activate the viral replication complex, a crucial site for viral replication (Melia et al., 2019). The development of ROs begins at the interface between the ER and Golgi, although the cellular markers of these membranes are swiftly lost (Nchoutmboube et al., 2015; Figure 3, bottom left). Considering that replication compartments are characteristic of all enteroviral infections, preventing their development may serve as a general anti-enteroviral therapeutic method (Laufman et al., 2019). According to one previous study, acyl-coenzyme is a metabolic enzyme, and is acyl-coenzyme A synthetase long-chain family member 4 (ACSL4), which participates in viral replication and organelle development. ACSL4 is essential for ferroptosis, while ferroptosis inhibitors reduce the viral load of CV-A6, indicating that ACSL4 is a potential antiviral target. It remains unknown whether ACSl4 performs the same function during E30 infection (Kung et al., 2022). Remarkably, mutant CV can replicate its genome in the absence of ROs through the Golgi apparatus when PI4KB is inhibited (Melia et al., 2017). However, few studies have focused on the role of RO during E30 replication, and it remains to be determined whether inhibiting RO production inhibits E30 replication.

The entire process of RNA replication can be loosely divided into two primary phases of initiation and extension (Pascal et al., 2020). After E30 is internalized, the genetic material is decapitated and released into the cytoplasm by FcRn, where it then serves directly as a protein encoding the mRNA-transduced virus. In addition, translation initiation is regulated by IRES via a cap-independent method (Belsham, 2009). Proteins that replicate RNA are among those that recruit RNA into the RO. According to studies on the replication of the oligonucleic acid virus family, the structure of the RNA in the UTRs and the coding area of the genome regulate the creation of the first viral proteins and the order of the negative-chain and positive-chain viral RNAs (Wang et al., 1999; Ogram and Flanegan, 2011). The full-length viral multiproteins were never found since they had been swiftly degraded by viral-encoded proteases, including co-processing and post-translational processing (cis and trans), particularly 2Apro, 3Cpro, and 3CDpro of EVs. Processing produces predominantly the precursor polyproteins P1 or P1-2A (depending on the major lytic sites at the N-terminal or C-terminal end of 2A), P2, and P3. Pro-P1/P1-2A is lysed, and capsid proteins (VP0, VP3, and VP1) are generated, whereas pro-P2 and P3 are transformed into their respective nonstructural proteins. In addition, processing generates a number of processing intermediates (e.g., 2 BC, 3AB, and 3CD), each of which plays a variety of roles in viral replication (Sasaki et al., 2012; Jackson and Belsham, 2021).

During the initial phase of E30 infection, viruses inhibit host cell cap-dependent translation (Jackson and Belsham, 2021). Their genome RNA is then replicated using a (−) RNA template that is transcribed and amplified at a low copy number prior to being packaged into a newly assembled virion and leaving the host cell via secretion or cell lysis (Rumlova and Ruml, 2018). AP-1 promotes the DNA entropion *in vitro*, a typical event in viral infections, according to previous research (Guo et al., 1996). It is unknown whether AP-1 plays a similar role in the replication of the E30 virus. The generation of viral particles does not require cellular lipids, according to metabolic investigations of E30 infection. Similar to E30 for EV-B, RNA-dependent RNA polymerase (RdRP) is the nonstructural protein required for genome replication (Rothe et al., 2010). Given that the RdRP protein is conserved throughout Picornaviridae (Whitton et al., 2005), its RNA is an ideal target for RNA interference (RNAi) to block virus replication. It has been reported that knockdown of DAF with RNAi results in only partial viral suppression, whereas shRdRP5 demonstrates potent inhibition of E30 in cell viability assays and the dual-expression vector scAAV2-SiDEx-shDAF2-shRdP5 has a strong inhibitory effect on E30 replication (Rothe et al., 2010). Overall, this provides insight into our ability to prevent the reproduction and subsequent spread of E30.

### 3.5. Maturation and release

In the nucleus and cytoplasm, newly synthesized E30 viral nucleic acids and structural proteins are assembled into progeny virus particles, which are then transferred from the inside to the exterior of the cell for release. Viruses interact with the cell membrane in the nucleus or cytoplasm, or they are assembled independently of the cell membrane, depending on the species. The majority of viruses are assembled through interactions between their structural proteins and the cell membrane (Rumlova and Ruml, 2018). Even though the assembly of mature particles belonging to the Picornaviridae has been studied for decades, very little is known about the mature release of E30. The current data reveals that this process involves sophisticated multiprotein cascade processing by viral-encoded proteases that construct a well-defined pentamer of VP0, VP3, and VP1 as intermediates (Tuthill et al., 2010). This study suggests that poliovirus (Bird and Kirkegaard, 2015a,b) and CV (Alirezaei et al., 2012) are released extracellularly via vesicle-mediated non-lytic virus transmission, whereas little is known about the release of mature E30, which may require specific interactions between E30 viral structural proteins and glycoproteins (Rumlova and Ruml, 2018). The final cellular separation of mature E30 from the cell may resemble the cellular mechanism used in the final cell division (releasing the daughter cell) (Rumlova and Ruml, 2018), known as the endosomal sorting complex (ESCRT). Hepatitis A virus, a member of the small ribonucleic acid virus family, interacts with ESCRT and is eliminated from the cell surface (Feng et al., 2013). It is currently unclear whether E30 undergoes a similar process. It has been demonstrated that some enteric viruses spread *in vitro* via vesicle virus clusters; however, it is unknown whether E30 is similar (Santiana et al., 2018). We therefore believe that E30, after entering the host cells, may also mediate viral transmission through vesicles and may simultaneously carry many viral particles into the CNS, causing significant harm. In addition, previous *in vitro* studies have demonstrated that certain EVs can boost viral replication via autophagy, including pancreatic acinar cell-specific autophagy, which is inhibited, and that CV replication *in vivo* is decreased (Alirezaei et al., 2012). It also remains unknown whether the same behavior occurs in the E30 virus. Therefore, additional research is required to determine the alterations that occur in host cells following virus invasion.

## 4. Conclusion and prospects

In summary, E30 interacts with DAF on the surface of the cell membrane, before entering cells via lipid raft protein, and ultimately binds to the uncoating receptor FcRn after entering cells. This causes conformational changes in the virus as well as the release of genetic material for reproduction. Replication requires replicator RO at the interface of the Golgi apparatus and endoplasmic reticulum. Once the mature virus bodies have been processed, assembled, and released, they can be either lytic or non-lytic (Figure 3). FcRn is necessary for the invasive cellular activities of E30, which are important for the comprehension of E30-induced CNS-associated illness pathways and serve as a guide for the creation of vaccines and therapies.

Recent research on post-invasion stages such as endocytosis, viral genomic assembly, and release, as well as the role of new receptor molecules on invasion, are not yet fully understood. Once E30 penetrates a comparable receptor, the important changes that occur in the host cell are unclear, therefore, additional research is required to understand the invasion process. In addition, further research is required to investigate the non-neurological problems caused by E30 as well as its connection to AFP and HMFP. It remains to be determined if the sites involved in the binding of DAF and FcRn to E30 can inhibit TRIO-RhoA signaling, if drugs and vaccines can be developed to prevent or treat AML in neonates, and if a broad spectrum of antiviral drugs or combination vaccines for other diseases can be developed based on FcRn characteristics.

## Author contributions

YL, JC, CW, and BY organized the content of the entire manuscript and wrote sections. YL, JC, and BY were responsible for the figures. YZ and ZL contributed substantially to the conception and design of the work. All authors contributed to the article and approved the submitted version.

## Funding

This work was funded by the National Key Research and Development Program of China (Project No. 2021YFC2302003) and Natural Science Foundation of Shandong Province (Grant Number: ZR2022QC209).

## Conflict of interest

The authors declare that the research was conducted in the absence of any commercial or financial relationships that could be construed as a potential conflict of interest.

## Publisher’s note

All claims expressed in this article are solely those of the authors and do not necessarily represent those of their affiliated organizations, or those of the publisher, the editors and the reviewers. Any product that may be evaluated in this article, or claim that may be made by its manufacturer, is not guaranteed or endorsed by the publisher.
